# Suicidality and/or Death-Related Thoughts in Health Workers After Pandemics: The Role of DYMERS

**DOI:** 10.3390/jcm14238343

**Published:** 2025-11-24

**Authors:** Antonio Urban, Elisa Cantone, Viviana Forte, Michela Atzeni, Stefano Lorrai, Mauro Carzedda, Gabriele Finco, Cesar Ivan Aviles Gonzalez, Sergio Machado, Roberta Montisci, Enzo Tramontano, Orsola Marra, Francesco Muscas, Fabrizio Bert, Giulia Cossu, Mauro Giovanni Carta

**Affiliations:** 1 Doctoral School in Capacity Building for Global Health, Department of Medical Sciences and Public Health, University of Cagliari, 09124 Cagliari, Italy; urban.azienda@gmail.com (A.U.); elisa.cantone@libero.it (E.C.); vivianaforte@gmail.com (V.F.); 2 Cagliari University Hospital, 09124 Cagliari, Italy; michela.atzeni93@gmail.com (M.A.); gabriele.finco@unica.it (G.F.); 3 Department of Medical Sciences and Public Health, University of Cagliari, 09124 Cagliari, Italy; stefanolorrai@live.it (S.L.); carzeddamauro@gmail.com (M.C.); rmontisci@unica.it (R.M.); orsola.marra@gmail.com (O.M.); muscas.franco@icloud.com (F.M.); maurogcarta@gmail.com (M.G.C.); 4 Department of Medicine, University of Enna ‘Kore’, 94100 Enna, Italy; cesar.aviles@unikore.it; 5 Instituto de Psiquiatria, Universidade Federal do Rio de Janeiro, Rio de Janeiro 22290-140, Brazil; secm80@gmail.com; 6 Department of Life and Environmental Sciences, University of Cagliari, 09124 Cagliari, Italy; tramon@unica.it; 7 Department of Public Health and Pediatrics Sciences, University of Torino, 10124 Torino, Italy; fabrizio.bert@unito.it; 8 Department of Nursing, Universidad Popular del Cesar, Cesar 200001, Colombia; 9 PhD Program in Tropical Medicine, Universidad Popular del Cesar, Cesar 200001, Colombia

**Keywords:** healthcare workers, death-related thoughts, depression, subthreshold symptoms, circadian rhythm dysregulation, healthcare workers, DYMERS, (dysregulation of mood, energy, and social rhythms)

## Abstract

**Background/Objectives**: Healthcare workers (HCWs) experienced marked psychological distress during and after the COVID-19 pandemic, including high levels of burnout, depression, and suicidal ideation. Italy, one of the first Western countries to be severely affected, recorded high mortality, even among healthcare staff. Emerging evidence suggests that suicidal ideation may also occur in the absence of major depressive episodes, possibly linked to subthreshold mood dysregulation and circadian rhythm disturbances, described within the construct of Dysregulation of rhythms and hyper-energy Syndrome (DYMERS). This study examined the prevalence of suicidal ideation and/or death-related thoughts among Italian HCWs, with particular attention to clinically relevant signs emerging in the absence of a full depressive episode. **Methods**: A cross-sectional analysis was conducted on 97 HCWs at the University Hospital of Cagliari, Italy, compared with a pre-pandemic community sample from the same region. Depressive symptoms and suicidal ideation or death-related thoughts were assessed with the Patient Health Questionnaire-9 (PHQ-9), considering their presence both in individuals with and without a depressive episode. Daily activation and energy were measured using item 10 of the 12-Item Short Form Health Survey (SF-12), while circadian rhythm dysregulation was evaluated with the Biological Rhythms Interview of Assessment in Neuropsychiatry (BRIAN). **Results**: HCWs showed a significantly higher prevalence of suicidal or death-related thoughts than the community sample (14.4% vs. 5.7%, *p* = 0.001; OR = 2.81, 95% CI 1.4–4.4). Notably, 8.2% of HCWs without probable depression reported such thoughts; given the very small number of events (n = 4), this estimate is exploratory, and inferential statistics should be interpreted with caution. Based on descriptive data, these individuals appeared to show higher perceived activation (SF-12, item 10) and rhythm dysregulation (BRIAN). This observation is exploratory and consistent with the DYMERS heuristic framework. **Conclusions**: Subthreshold symptoms and DYMERS may represent critical risk factors in suicide prevention strategies for HCWs.

## 1. Introduction

Healthcare workers (HCWs) experienced substantial increases in burnout, depression, anxiety, PTSD, sleep disturbances, and emotional exhaustion during the COVID-19 pandemic and across its aftermath [1,2,3,4]. The pandemic’s impact on healthcare workers and on the healthcare system has been severe in Italy. Italy was among the hardest-hit countries in the initial phases of the pandemic, with high infection rates and a high mortality rate [5,6]. Italy was the first Western nation to be affected, and mortality was particularly high, even among healthcare workers [7,8,9,10,11].

Secondly, the impact of the pandemic undermined the belief—widespread among healthcare workers, politicians, and citizens—that the Italian healthcare system was one of the best in the world and would therefore respond without the problems seen in other countries [8,11]. The loss of this confidence generated a sense of impotence and unpreparedness and led to feelings of frustration and increased demands among healthcare workers [12,13,14]. The stressful situation to which healthcare workers were subjected raised the issue of suicide risk [15]; in Italy, media reported suicides among health workers during the pandemic with an impact on the community [16]. An umbrella review analyzing 400 international studies from 2004 to 2023 on healthcare worker burnout and suicide risk found an upward trend in relation to the impact of the pandemic [17]. An international multicenter study also highlighted that the pandemic was associated with an increase in suicidal ideation in healthcare professionals [18]. A large review of the international literature found that about 17% of physicians had suicidal ideation and ~1% had attempted suicide, though rates varied geographically and increased after the pandemic [15]. Studies on suicide risk in healthcare professionals have also been conducted in Italy, which reported an increase linked to the pandemic [8,19]. A community survey found that thoughts of death and/or suicide were relatively common even among individuals who did not exhibit symptoms consistent with a full diagnosis of depressive disorder [20]. To explain this phenomenon, it has been hypothesized that syndromes in the bipolar spectrum with subthreshold symptoms compared with depressive episodes, insufficiently studied and not included in the official nosology—such as recurrent brief depression [21,22,23,24]—and the Syndrome of dysregulation of rhythms and hyper-energy, “DYMERS,” which was recently identified [25], may play a role. Recent evidence has also emphasized the contribution of genetic and neurobiological mechanisms to suicidal behavior, involving stress-response and immune-modulation pathways [26,27,28]. These findings suggest a multifactorial vulnerability framework in which biological susceptibility interacts with rhythm dysregulation and psychosocial stressors, potentially predisposing healthcare workers to subthreshold affective dysregulation and suicidal ideation. Elements of rhythm dysregulation/hyperarousal have been detected in healthcare workers with significant levels of burnout, especially during the COVID pandemic [29]. It could be interesting to investigate whether suicidal thoughts can be detected in healthcare workers experiencing work-related stress in the absence of a depressive episode, i.e., in the presence of “subthreshold” depressive symptoms. This aspect is relevant for both support planning and preventive intervention. Given that suicidal ideation is a well-established risk factor for suicide [30], the aim of this study is to assess the prevalence of suicidal ideation in a sample of highly stressed healthcare workers and to explore its role and possible occurrence among individuals with subthreshold depressive symptoms.

Our hypothesis is to investigate, within this study, the presence of depressive episodes among health professionals, with particular emphasis on identifying clinically relevant warning signs such as thoughts of death or suicide, even in the absence of a full depressive episode. Such manifestations may serve as important early indicators and could inform the development of targeted preventive interventions for professionals. Furthermore, the study aims to explore whether features consistent with a syndrome of rhythm dysregulation and hyperactivity—characteristic of DYMERS—together with subthreshold depressive symptoms, may function as concomitant risk factors in the population under investigation, or may themselves represent concomitant features of the same rhythm dysregulation syndrome.

## 2. Materials and Methods

### 2.1. Design

This is a cross-sectional observational design, on a sample of health professionals from the outpatients centers of the University Hospital of Cagliari, Italy.

### 2.2. Sample

A voluntary sample comprising health professionals working in five outpatient units (pain therapy, oncology, dermatology, endocrinology, and cardiology) of a single hospital in Sardinia, Italy, was recruited. The study was approved by the Ethics Committee of the Region of Sardinia on 18 September 2025, under protocol number 25569. Participant recruitment took place between 7 October and 14 October 2025.

Participants were eligible for inclusion if they met the following criteria: (i) age ≥ 18 years (18 years + 1 day); (ii) either sex; (iii) provision of written informed consent; and (iv) active professional engagement in healthcare within one of the following categories: physicians, nurses, psychologists, healthcare assistants (OSS), biologist, social workers, rehabilitation professionals, educators, as well as postgraduate trainees in psychology and medical specialties.

In total, 97 health professionals participated and were included in the present study sample. Two individuals (one medical doctor and one nurse) declined to participate due to time constraints, representing 2.06% of those invited.

All questionnaires were fully completed and included in the analysis, with no cases excluded due to missing data.

The comparison was conducted with a population-based survey carried out in Sardinia (Italy) prior to the COVID-19 pandemic using computer-assisted telephone interviewing (CATI). The sample, drawn from the same geographical area, was stratified by province, sex, and age group, and included over 2300 eligible individuals. Standardized instruments, including case vignettes and the Patient Health Questionnaire (PHQ-9), were administered to assess depressive symptoms, help-seeking attitudes, and related factors [31].

### 2.3. Instruments

Health professionals who agreed to participate in the study, after providing informed consent, completed the study instruments.

The instruments adopted for the study include:

1. Sociodemographic and Work-Related Data Sheet

A tool designed to collect information on general work-related and demographic characteristics (age, gender, workplace, and occupational role). To preserve anonymity—since cross-referencing data could potentially allow identification—less common professions (e.g., social worker, nutritionist, dentist, or security officer) were grouped into broader categories. For the same reason, no user diagnoses were recorded.

2. Patient Health Questionnaire-9 (PHQ-9)

The Patient Health Questionnaire-9 [32], Italian version [33,34], was employed in the main study as a self-administered instrument for screening depressive episodes in the sample. This nine-item questionnaire is specifically designed to detect depressive episodes and to assess and monitor the severity of depressive symptoms, incorporating all the criteria from the Diagnostic and Statistical Manual of Mental Disorders (DSM) [35] for the diagnosis of a major depressive episode into a concise self-report format. In the present analysis, a cut-off score greater than 9 was adopted to identify a possible depressive episode (as this is a screening tool), in line with a previous study conducted on health professionals and service users [36]. To identify the presence of thoughts related to death or suicide, participants who scored at least 1 on the PHQ-9 item assessing such thoughts on a five-point Likert scale (“Thoughts that you would be better off dead or of hurting yourself in some way”) were selected.

3. Short-Form Health Survey (SF-12)

The Short-Form Health Survey [37] is an instrument developed to assess health-related quality of life (H-QoL), encompassing both physical and psychological dimensions. It consists of 12 items that evaluate the extent to which an individual perceives their health as affecting daily activities. Higher scores indicate a greater perceived quality of life [38]. In this study, only item 10 of the SF-12 was considered, which assesses the level of daily activation or energy. The standardization of this single-item screener against the Mood Disorder Questionnaire (MDQ) [39] which specifically captures aspects of excessive energy or activity during hypomanic episodes—has already been conducted in previous studies, which found a strong association between the single SF-12 item and the MDQ (sensitivity ≈ 0.72; specificity ≈ 0.70) and with MDQ items related to excessive energy and activity [40]. While this single-item approach offers a pragmatic and efficient proxy for perceived energy in applied settings, we acknowledge that it cannot fully capture the multidimensional complexity of the energy construct. This functional dimension has also been linked to impaired quality of life in individuals screening positive on the Mood Disorder Questionnaire, supporting the DYMERS hypothesis [41].

4. Biological Rhythms Interview of Assessment in Neuropsychiatry (BRIAN)

The Biological Rhythms Interview of Assessment in Neuropsychiatry (BRIAN) [42] is a tool consisting of 18 items designed to evaluate disturbances in biological rhythms. It covers key domains such as sleep, daily activities, eating patterns, and social rhythms. Higher total scores indicate greater dysregulation of biological rhythms [43].

### 2.4. Statistical Analysis

Data were analyzed using the chi-square test, with Yates’ correction applied when required. Given the limited sample size, continuous variables were excluded, and the analysis focused exclusively on dichotomous outcomes: the presence or absence of a depressive episode and the presence or absence of death- or suicide-related thoughts, as derived from the assessment instruments. Medians of item 10 of the SF-12 and BRIAN total scores were then calculated in relation to the PHQ-9 median among participants who did not screen positive on the PHQ-9 but reported thoughts of death or suicide. Since we needed to use the community survey data in comparison with a sample of health workers, we extracted only data relating to the 25–70 age group (25 was the minimum age of workers and 70 was the maximum retirement age for health workers at the time of the survey). The analysis was further conducted in the four age groups divided by sex and age (25–44 and 45–69). To minimize sparse-cell bias in contingency analyses, dichotomous outcomes were used throughout. A depressive episode was defined as PHQ-9 > 9 (screening threshold previously adopted in health-worker samples) [36]. Death/suicide-related thoughts were coded present when PHQ-9 item 9 ≥ 1. Group comparisons were stratified by sex and age bands (25–44 vs. 45–69), mirroring the community survey structure and the working/retirement age range used for matching. Inferential tests used χ^2^ with Yates’ correction when expected counts were <5; cells with very small n were treated as descriptive only and interpreted cautiously. SF-12 item 10 (energy) and BRIAN total were summarized descriptively (medians) and were not entered into inferential tests. The statistical analyses were conducted using Stata software, version 17.0

### 2.5. Ethics

The study was approved by the Independent Ethical Committee of Regione Sardegna, Italy, on 18 September 2025, under protocol number 25569. The research was carried out in agreement to the Declaration of Helsinki [44]. The informed consent signed by participants explained that all data for the study would be collected through anonymous records and that each participant would be free to abandon the research if they wanted in any moment. Any information and explanations about the study could be asked using one dedicated telephone number and e-mail address.

## 3. Results

The study sample consisted of 97 healthcare workers. The participants’ mean age was 43.8 years (SD = 12.55). Regarding relationship status, 50.5% of participants were single (n = 49), 40.2% were married (n = 39), 5.2% were divorced (n = 5), 1.0% were widowed (n = 1), and 3.1% reported being in other relationships such as cohabiting or engaged (n = 3). The majority were medical doctors (n = 54; 55.7%), among whom 9 participants (9.3% of the total sample; 16.7% of physicians) were medical residents or trainees. Nurses constituted 30 participants (30.9%). The remaining participants were operating support staff (OSS) (n = 11; 11.3%) and nutrition biologists (n = 2; 2.1%). This composition reflects a diverse representation of healthcare roles within the clinical setting. Table 1 shows the frequency of thoughts of death and suicidal ideation in a sample of health workers and the comparison with the community sample. In general, the health worker sample showed a higher frequency (14.4% vs. 5.7%, *p* = 0.001). A higher frequency of thoughts of death and suicidal ideation in the health worker sample persists in the four cells by sex and age (25–44 and 45–69), however in none of these cells was a statistically significant difference reached. If the data were grouped by sex, only women showed a higher frequency than the control group (16.7% vs. 6.4%, *p* = 0.005).

Table 2 shows the frequency of thoughts of death and suicidal ideation in people with a depressive episode (PHQ > 9) from the study sample and the comparison with the frequency of thoughts of death and suicidal ideation in people with a depressive episode (PHQ > 9) from the community sample. No statistically significant differences were found in the overall sample, in the subsamples by gender, and in the four subsamples by age and gender. In all these analyses, however, a tendency toward a lower frequency was observed in the general population sample. Given the limited sample size and the very small counts observed in some subgroup cells (particularly among men), the relevant comparisons should be considered descriptive only. ORs, confidence intervals, and *p*-values are reported for completeness and do not support robust inferences.

Table 3 shows the frequency of death and/or suicidal thoughts in the subsample of people with suicidal ideation without depressive episode (people with subthreshold depressive symptoms) in comparison with the community survey subjects in a similar condition. The frequency in the total sample is significantly higher among health workers (8.2% vs. 2.5%, *p* = 0.017). In the analysis by gender, the difference between health workers and the community sample increases in males (20% vs. 2.4%, *p* = 0.017) but does not reach statistical significance in females. The analysis in the 4 cells divided by age and gender is affected by the low frequency of observations; however, among young males, a statistically significant higher frequency is found among health workers (50% vs. 2.2%, *p* < 0.0001); however, this comparison is based on a very small number of cases (2/4) and should be considered purely descriptive, without statistical inferences.

Table 4, useful only at a purely descriptive level given the small residual sample size, which did not allow for further analysis, shows the characteristics of hyperactivity and rhythm dysregulation of the 4 people with death and/or suicidal thoughts without depressive episode. The score on the single item of SF-12 places them in that area of non-continuous and periodic hyperactivation (scores 3-4-5) [40] which has been hypothesized to be typical of DYMERS. The rhythm dysregulation is highly dysfunctional for all of them, well above the score (22.22) of the community sample used for the validation of the BRIAN instrument [43].

## 4. Discussion

### 4.1. Main Findings, Empirical Contribution, and Integration with Prevention Frameworks

A previous study based on the same database highlighted a worrying prevalence of thoughts of death and/or suicidal ideation among a sample of Italian healthcare professionals in the post-pandemic period, significantly higher than in the general population. However, the present secondary analysis showed that the specific frequency of thoughts of death and/or suicidal ideation in people with depression among the sample does not differ from that of people with a depressive episode of the community sample. Therefore depressive episodes in health workers do not have a higher frequency of suicidal ideation than depression in the community sample. Conversely, this study found a higher frequency of death and/or suicidal ideation in a relevant portion of the health worker sample with subthreshold depressive symptoms without a depressive episode (8.2%). Our primary empirical contribution is the indication that suicidal ideation can emerge among HCWs presenting subthreshold depressive symptoms, i.e., in the absence of a full depressive episode. This finding suggests that greater attention to subthreshold mood symptoms in HCWs—beyond major depression—could be valuable within occupational mental-health monitoring and secondary prevention, particularly for groups exposed to sustained or acute work-related stress. A numerically higher rate was observed among men, a group generally considered at higher risk for suicide [45].; however, this comparison is based on very small cell counts (n = 10 in total) and should be interpreted as descriptive only, pending confirmation in larger samples.

### 4.2. Multifactorial Framework and Broader Determinants

The NHS Health and Wellbeing Framework promotes early recognition of psychological distress among healthcare workers, with attention to gender-related risk patterns and occupational stressors such as burnout and moral injury. It advocates supportive organizational cultures, access to psychological help, and structured postvention plans to reduce suicide risk and enhance workforce wellbeing [45].

This work does not aim to understate the multiplicity of well-documented factors that contribute to suicide risk [46]. On the biological side, recent evidence indicates genetic and gene–environment contributions [26,27,28]; at the sociocultural level, differences in norms, beliefs, and stigma shape both symptom expression and help-seeking behavior [47]. Macro-social factors such as economic vulnerability and structural disadvantage are associated with increased risk [48,49,50], as are substance-use disorders, which can amplify impulsivity and psychological distress [51,52,53]. Loneliness—conceived both as objective isolation and as the subjective perception of disconnection—also emerges as a cross-cutting predictor of suicidal thoughts and behaviors [54,55,56]. Finally, educational attainment influences health literacy, service access, and coping strategies, thereby influencing trajectories of risk and protection [57]. Within this multifactorial framework, our findings draw clinical attention to dimensions often under-recognized in work-related stress contexts, such as episodic hyperarousal and the dysregulation of sleep–wake and social rhythms.

Previous research on suicidality among healthcare workers during the COVID-19 period has documented elevated risk and highlighted depression, PTSD, and burnout as key correlates [15,17,19], with multi-country time-series work charting pandemic-related fluctuations in suicidal ideation [18]. In contrast, the present study explores the occurrence of suicidal ideation in the absence of a full depressive episode, emphasizing the role of subthreshold mood symptoms and biological-rhythm dysregulation as potential vulnerability markers.

### 4.3. Possible Mechanisms Underlying Suicidal Ideation in HCWs in the Absence of a Full Depressive Episode

Recurrent brief depression (RBD) is a disorder in the bipolar spectrum notoriously more frequent in men [22] that does not meet the threshold of a depressive episode because the symptoms are fleeting and last only a few days with frequent brief recurrence [21,22]. This syndrome is difficult to identify, especially using screening tools that only assess the last week. However, RBD exposes the individual to an even higher risk of suicide than major depression [23,24,58]. A recent systematic review and meta-analysis on 52 studies found that social rhythm disruption showed an association with suicidal thoughts/behaviors (Hedges g ≈ 0.52 for STB; 0.57 for ideation), and even circadian rhythm sleep–wake disorders—especially delayed sleep–wake phase—were associated with higher risk [59] particularly among men [60] who have greater difficulty expressing distress or seeking help [61].

The role of rhythm dysregulation is consistent with what has been observed longitudinally in adolescents [62] and in clinical samples involving various mood disorder pictures [63,64,65]. A robust body of research has shown that in stress syndromes, rhythm dysregulation is associated with hyperarousal and activation [66,67,68]. These findings have been synthesized in the construct of the Dysregulation of Mood, Energy, and Social Rhythms Syndrome (DYMERS) [25]. Although in our small sample all four individuals with suicidal ideation in the absence of a full depressive episode presented features consistent with hyperactivation and rhythm dysregulation, this pattern must be regarded as purely descriptive. Given the very limited number of observations, it cannot be interpreted as empirical evidence but rather as an exploratory signal consistent with the DYMERS hypothesis. It is plausible that circadian misalignment contributes to suicidal vulnerability via effects on sleep–wake stability, emotion regulation, and physiological arousal, potentially reducing cognitive control and increasing impulsivity [59,63,64,65]. These alterations might heighten stress sensitivity and lower resilience, even without a full depressive episode.

### 4.4. Implications and Concluding Remarks

In this perspective lies the heuristic hypothesis of DYMERS, which integrates mood, energy, and social rhythms as a cluster of dysregulation with potential relevance for secondary prevention [25]. However, in this study such indications should be viewed as exploratory and not as confirmatory evidence, given the limited subgroup sizes and descriptive nature of the underlying data. Particularly noteworthy in our sample is the profile observed among men, though this observation—based on very small numbers—should also be interpreted cautiously and considered hypothesis-generating. Taken together, these elements do not contradict established genetic, cultural, and socioeconomic determinants; rather, they complement them by indicating that—in populations professionally exposed to chronic loads and acute peaks of stress—attention to episodic hyperarousal and misaligned rhythms may offer heuristic insights and potential avenues for future investigation, which could ultimately inform more timely and individualized prevention strategies. Although preliminary, these findings tentatively suggest that greater attention to subthreshold mood symptoms—beyond major depression—could be considered within occupational mental-health surveillance for HCWs, particularly in high-stress contexts, as part of a broader preventive reflection.

## 5. Limitations

This study has several limitations. First, the cross-sectional design prevents any inference of causality [69] between subthreshold depressive symptoms, circadian dysregulation, and suicidal ideation. Second, the reliance on self-report measures such as the PHQ-9 may underestimate transient or episodic mood symptoms like recurrent brief depression, which often escape weekly assessment tools. On the other hand, as with all screening tools, while they provide a concise and sustainable way to capture a snapshot of a clinical phenomenon, they cannot replace proper diagnostic instruments. Moreover, self-reported measures are susceptible to bias with potential underestimation/overestimation of symptoms and misleading conclusions [70]. Another limitation concerns the use of a single SF-12 item (item 10) as a proxy measure for perceived energy. Although this item has shown acceptable sensitivity and specificity when compared with the Mood Disorder Questionnaire [40], a single question cannot fully capture the multidimensional nature of energy and activation, and future studies should employ more comprehensive instruments to assess this construct. In addition, no data were available on potential confounders such as prior psychiatric history or workload intensity, and the study did not control for institutional differences between care settings. Only the latter can confirm whether the presence of the sign investigated in the present study can indeed be considered attributable to a structured disorder. Furthermore, the low sample size within specific subgroups (e.g., males with subthreshold symptoms) may limit statistical power and generalizability [71]. Moreover, voluntary participation may have introduced selection bias, as those experiencing greater distress might have been more motivated to take part. Additionally, there may have been differences in the results based on the socio-demographic and role composition of the sample which, however, with such a small number, could not be controlled or estimated.

Finally, the study is based on a single regional sample, which may not reflect the broader Italian healthcare population.

## 6. Conclusions

This study highlights the importance of considering factors beyond diagnosable depressive episodes when addressing suicide risk among healthcare professionals. The observation of suicidal ideation in individuals without major depression—particularly among men—suggests that subthreshold mood dysregulation and rhythm disturbances may warrant further investigation. These exploratory findings are consistent with the heuristic framework of the DYMERS construct, which may offer a useful lens for understanding post-pandemic psychological vulnerabilities. However, given the small sample size and descriptive nature of the analyses, these results should be regarded as preliminary. Future research, based on larger and longitudinal samples, is needed before drawing firm conclusions or recommending specific preventive interventions.

## Figures and Tables

**Table 1:** Thoughts of death and suicidal ideation in a sample of 97 health workers in comparison with the community sample.

Group	Age: 45–69 N (%)	Age: 25–44 N (%)	Total (by Sex) N (%)
Women (Community)	22/304 (7.2%)	16/268 (5.9%)	38/572 (6.6%)
Women (Health Workers)	6/35 (17.1%)	5/33 (15.1%)	11/68 (16.7%)
Chi Square 1df	1.168 *p* = 0.280 OR = 1.71, 95% CI [0.6–4.6]	3.816 *p* = 0.051 OR = 2.81, 95% CI [1.0–8.2]	7.818 *p* = 0.005 OR = 2.71, 95% CI [1.3–5.6]
Men (Community)	13/286 (4.1%)	13/273 (4.8%)	26/559 (4.6%)
Men (Health Workers)	2/11 (18.2%)	1/18 (5.5%)	3/29 (10.3%)
Chi Square 1df	(Yates) 1.756 *p* = 0.185 OR = 4.67, 95% CI [0.9–23.8]	(Yates) 0.0001 *p* = 0.999 OR = 1.18, 95% CI [0.1–9.5]	(Yates) 0.885 *p* = 0.347 OR = 2.36, 95% CI [0.6–8.3]
Total (by age) Community	35/590 (5.9%)	29/541 (5.4%)	64/1131 (5.7%)
Health Workers	8/46 (17.4%)	6/51 (11.8%)	14/97 (14.4%)
Chi Square 1df	8.889 *p* = 0.003 OR = 3.34, 95% CI [1.4–7.7]	3.436 *p* = 0.064 OR = 2.35, 95% CI [0.9–6.0]	11.563 *p* = 0.001 OR = 2.81, 95% CI [1.4–4.4]

Legend: OR = Odds Ratio; CI = Confidence Interval; df = degrees of freedom.

**Table 2:** Thoughts of death and suicidal ideation in people with depressive episode (PHQ > 9) comparing a sample of 97 health workers with a community sample.

Group	Age: 45–69 N (%)	Age: 25–44 N (%)	Total by Sex N (%)
Women (Community)	15/31 (48.4%)	10/26 (38.5%)	25/57 (43.8%)
Women (Health Workers)	6/16 (37.5%)	3/13 (23.1%)	9/29 (31.0%)
Chi Square 1df	0.506 *p* = 0.477 OR = 0.64, 95% CI [0.2–2.2]	0.923 *p* = 0.337 OR = 0.48, 95% CI [0.1–2.1]	1.323 *p* = 0.250 OR = 0.58, 95% CI [0.2–1.5]
Men (Community)	7/13 (53.8%)	6/14 (42.9%)	13/27 (48.1%)
Men (Health Workers)	0/1 (0%)	1/4 (25%)	1/5 (20%)
Chi Square 1df	(Yates) 0.006 *p* = 0.936 OR = 0.54, 95% CI [0.2–1.7]	(Yates) 0.004 *p* = 0.948 OR = 0.44, 95% CI [0.1–5.4]	(Yates) 0.455 *p* = 0.500 OR = 0.27, 95% CI [0.1–2.7]
Total by age Men + Women Community	22/44 (50%)	16/40 (40%)	38/84 (45.2%)
Total by age Health Workers Sample (M + W)	6/17 (35.3%)	4/17 (23.5%)	10/34 (34%)
Chi Square 1df	1.068 *p* = 0.301 OR = 0.54, 95% CI [0.2–1.7]	0.769 *p* = 0.683 OR = 0.77, 95% CI [0.2–2.7]	1.655 *p* = 0.198 OR = 0.58, 95% CI [0.2–1.3]

Legend: OR = Odds Ratio; CI = Confidence Interval; df = degrees of freedom. Analyses in cells with very small counts (e.g., n < 5) are descriptive only. ORs and *p*-values, when reported, are presented for completeness but do not support robust inferences.

**Table 3:** Thoughts of death and suicidal ideation in individuals without a depressive episode (identified as potential subthreshold depression), compared between a sample of 97 health workers and a community sample.

Group	Age: 45–69 N (%)	Age: 25–44 N (%)	Total by Sex N (%)
Women (Community)	7/273 (2.6%)	6/242 (2.5%)	13/515 (2.5%)
Women (Health Workers)	0/19 (0%)	2/20 (10%)	2/39 (5.1%)
Chi Square	(Yates) 0.0001 *p* = 0.999 OR = 0, 95% CI [0-NaN]	(Yates) 1.446 *p* = 0.229 OR = 4.37, 95% CI [0.8–23.2]	(Yates) 0.206 *p* = 0.650 OR = 2.09, 95% CI [0.4–9.6]
Men (Community)	6/273 (2.2%)	7/259 (2.7%)	13/532 (2.4%)
Men (Health Workers)	2/4 (50%)	0/6 (0%)	2/10 (20%)
Chi Square 1df	(Yates) 17.336 *p* < 0.0001 OR = 44.5, 95% CI [5.3–370.8]	(Yates) 0.0001 *p* = 0.999 OR = 0, 95% CI [0-NaN]	(Yates) 5.665 *p* = 0.017 OR = 9.98, 95% CI [1.9–51.7]
Total by age Men + Women Community	13/546 (2.4%)	13/501 (2.6%)	26/1047 (2.5%)
Total by age Health Workers Sample (M + W)	2/23 (8.7%)	2/26 (7.7%)	4/49 (8.2%)
Chi Square 1df	(Yates) 1.410 *p* = 0.235 OR = 3.9, 95% CI [0.8–18.4]	(Yates) 0.845 *p* = 0.358 OR = 3.1, 95% CI [0.7–14.6]	(Yates) 5.672 *p* = 0.017 OR = 3.49, 95% CI [1.2–10.4]

Legend: OR = Odds Ratio; CI = Confidence Interval; df = degrees of freedom. Analyses in cells with very small counts (e.g., n < 5) are descriptive only. ORs and *p*-values, when reported, are presented for completeness but do not support robust inferences.

**Table 4:** Individual with death and/or suicidal ideation with depressive subthreshold symptoms (dysregulation of rhythms and activation profile).

	SF-12 (Item 10, Activation)	BRIAN	PHQ-9 Score	Age Range
1	4	50	8	Female 25–44
2	5	38	3	Female 25–44
3	3	46	8	Male 45–69
4	4	36	5	Male 45–69
Median	4	43	7	

Legend: SF-12 = 12-Item Short Form Health Survey; PHQ-9 = Patient Health Questionnaire-9; BRIAN = Biological Rhythms Interview of Assessment in Neuropsychiatry.

## Data Availability

The datasets of this study will not be publicly available due to individual privacy rules.
